# Risk Factors for Recurrent Vitreous Hemorrhage in Type 2 Diabetes Mellitus Patients after Posterior Vitrectomy

**DOI:** 10.3390/jcm12082989

**Published:** 2023-04-20

**Authors:** Marc Baget-Bernaldiz, Pedro Romero-Aroca, Alejandra Mira-Puerto, Angel Bautista-Perez, Immaculada Roca-Borrut, Montse Vizcarro, Raul Navarro-Gil, Monica Llagostera-Serra

**Affiliations:** 1 Ophthalmology Service, Hospital Universitario Sant Joan de Reus, 43204 Reus, Spain; 2 Department of Medicine and Surgery, Faculty of Medicine and Health Sciences, Universitat Rovira i Virgili, 43201 Reus, Spain; 3 Pere Virgili Institute for Health Research (IISPV), 43204 Reus, Spain

**Keywords:** proliferative diabetic retinopathy, recurrent vitreous hemorrhage, diabetes duration, anemia, posterior vitreous, retinal laser photocoagulation

## Abstract

(Background) The aim of this study was to determine the factors related to recurrent vitreous hemorrhage (RVH) in a sample of proliferative diabetic retinopathy (PDR) patients. (Methods) This was a retrospective, review-based study. We studied 183 eyes from 121 type 2 diabetes patients with PDR. We recorded the duration of diabetes, history of hypertension, retinal photocoagulation status, posterior vitreous status, mean HbA1c and hemoglobin levels, renal function, and systemic complications associated with diabetes. We also recorded surgical variables—the presence of tractional retinal detachment, the application of segmentation and diathermy on fibrovascular proliferative tissue, and the use of silicone oil—to study which independent variables were significantly related to the presence of RVH. (Results) The duration of diabetes (*p* = 0.028), hemoglobin level (*p* = 0.02), status of the posterior vitreous (*p* = 0.03), retinal photocoagulation status (*p* = 0.002), and the presence of tractional retinal detachment (*p* = 0.03) were significantly associated with the presence of RVH. On the other hand, the use of diathermy was associated with fewer RVH events (*p* < 0.005). In addition, patients with diabetic polyneuropathy, myocardial infarction, and ischemia in the lower limbs exhibited more vitreous hemorrhage events (*p* < 0.001). (Conclusions) Patients with PDR and a longer diabetes duration, anemia, attached posterior vitreous, deficient retinal photocoagulation, and prior cardiovascular events were more prone to RVH.

## 1. Introduction

Diabetic retinopathy (DR) is the leading cause of visual loss in the working-age population in developed countries [1]. This is mainly due to ocular complications, such as diabetic macular edema (DME) and vitreous hemorrhage. The latter occurs in patients with proliferative diabetic retinopathy (PDR). The most established risk factors related to PDR development are the duration of diabetes [2,3,4], the degree of metabolic control of the disease, and blood pressure [5]. With respect to the eye, the presence or absence of posterior vitreous detachment (PVD) is probably the most important feature in determining retinal neovascularization in patients with PDR [6].

Despite the use of anti-vascular endothelial growth factor (anti-VEGF) drugs or lasers for the treatment of PDR, vitrectomy is still required in up to one-third of eyes with PDR mainly due to the occurrence of non-clearing vitreous hemorrhage [7,8]. In complicated cases with extensive fibrovascular proliferation or tractional retinal detachment, the bimanual technique is often applied to perform segmentation and delamination in order to free the retina, along with the use of silicone oil to prevent early rebleeding and allow time for the new vessels to heal [9]. However, one of the most common postoperative complications is the presence of recurrent vitreous hemorrhage (RVH), which may cause visual impairment and require re-operation [10]. The most common causes of RVH after vitrectomy are fibrovascular ingrowth at the sclerotomy sites, the formation of recurrent fibrovascular in the retina, and insufficient retinal photocoagulation [11]. It is quite common for diabetic patients to take anticoagulants or antiplatelet drugs in an effort to prevent cardiovascular events, although their role in vitreous hemorrhage is still inconclusive [12,13,14].

The aim of this study was to investigate which clinical and surgical variables were related to the presence of RVH in a population of patients diagnosed with PDR who had previously undergone 25-gauge posterior vitrectomy due to the occurrence of a first episode of vitreous hemorrhage.

## 2. Materials and Methods

### 2.1. Study Design

We evaluated 183 eyes from 121 T2DM patients. All were sampled from a reference population of Hospital Universitari Sant Joan de Reus, Tarragona, Spain. This study was part of our line of research on DR described elsewhere. We conducted a retrospective review of all T2DM patients who had undergone standard three-port pars plana vitrectomy (PPV) for non-clearing vitreous hemorrhage caused by PDR at our hospital.

### 2.2. Ethical Adherence

This study adhered to the legal requirements of our local ethics committee (approval CEIM 028/2018), in accordance with the revised guidelines of the Declaration of Helsinki. Informed consent was obtained from all participants in the study.

### 2.3. Inclusion Criteria

All patients with T2DM diagnosed with PDR in our health care areas, who presented with vitreous hemorrhage that needed vitreoretinal surgery between 1 June 2010 and 30 June 2020, were eligible for inclusion in this study.

### 2.4. Exclusion Criteria

Patients with any previous type of retinal vascular occlusion, with proliferative retinopathy of causes other than diabetes, and who had previously undergone operations for other retinal conditions were not eligible for inclusion in this study.

### 2.5. Methods

#### 2.5.1. Clinical and Surgical Variables Studied

All eyes were assessed by fundus examination, optical coherence tomography (OCT), and fluorescein angiography (FA) to ensure the presence of PDR after PPV was performed. All eyes underwent ocular ultrasonography to evaluate further details, such as the presence of the vitreous attached to the macula or to rule out tractional retinal detachment.

We conducted a comprehensive review of the electronic health report for all T2DM patients, including age, gender, DM treatment (insulin or oral antidiabetic drugs), DM duration, and blood pressure. We recorded the body mass index (BMI), estimated glomerular filtration rate (eGFR), urine albumin to creatinine ratio (UACR), and mean HbA1c and hemoglobin levels one year before being diagnosed with PDR and throughout the study period. We identified patients treated with anticoagulant or antiplatelet drugs and those diagnosed with chronic obstructive pulmonary disease (COPD). Finally, we identified patients who had suffered polyneuropathy, stroke, ischemic cardiopathy, or ischemia in the lower limbs.

All eyes were evaluated according to the status of the posterior vitreous using ultrasonography and/or intraoperatively. All eyes were classified into two subgroups depending on whether the vitreous was initially attached to the posterior pole. In addition, we recorded patients who had previously been treated with ranibizumab for macular edema. Finally, we recorded eyes that had previously undergone retinal laser photocoagulation before the first vitrectomy, for which we quantified the number of retinal laser sessions applied. A laser session was considered if there were at least 250 laser spots.

We recorded some intra-surgical variables such as the presence of tractional retinal detachment, the application of segmentation and delamination, and diathermy on the fibrovascular proliferative tissue of the retina. Additionally, we recorded the occurrence of retinal tears during each vitrectomy.

We classified all eyes with PDR into two groups depending on the behavior of the vitreous hemorrhage. The first group was those with eyes that had bled only once (SVH); the second group was those with eyes that had bled more than once (RVH). In addition, we further classified them into two subgroups depending on the time of recurrence. Early recurrence was considered within 15 days after PPV; late recurrence was after this period.

The severity of all vitreous hemorrhage episodes was scored on a 5-point scale according to Lieberman et al. [15], as follows: grade 0 (no vitreous hemorrhage); grade 1 (minimal vitreous hemorrhage and optic disk and retinal vessels are clearly visible); grade 2 (mild vitreous hemorrhage and most of the optic disk and retinal vessels are visible); grade 3 (moderate vitreous hemorrhage and optic disk or retinal vessels are barely visible); and grade 4 (severe vitreous hemorrhage and too dense to enable visualization of the optic disk).

In our study, all eyes in both groups that showed grades 3 or 4 vitreous hemorrhage and did not clear after 2 months underwent PPV along with additional retinal laser photocoagulation.

#### 2.5.2. Surgical Technique

All operations were performed under peribulbar anesthesia by four vitreoretinal surgeons who had more than 10 years of experience, using the Bausch&Lomb Stellaris PC (Bausch & Lomb, Rochester, NY, USA). Each surgery was carried out by two surgeons. A wide-angle contact indirect viewing system with a built-in image inverter was used in all cases. All patients underwent 25-gauge standard three-port PPV. In the first vitrectomy, the vitreous gel was removed as peripherally as possible, both in phakic and pseudophakic eyes, with the help of the second surgeon who performed scleral indentation to achieve better visualization. At the same time, panretinal laser photocoagulation was completed or performed. In cases where the fibrovascular proliferative tissue caused tractional retinal detachment, the bimanual technique was applied to perform segmentation and delamination in order to free the retina. In most of these cases, endodiathermy and silicone oil were used to avoid early rebleeding or retinal detachment due to the formation of more than one iatrogenic hole. At the end of the procedure, the cannulas were carefully removed, and all sclerotomies were sutured to avoid postoperative ocular hypotony. In all cases, intravitreal ranibizumab was administered at the end of surgery.

### 2.6. Statistical Analysis

Data were analyzed using SPSS (software IBM^®^ SPSS^®^ version 25.0, IBM Corp., Armonk, NY, USA). In this study, the dependent variable was the presence of recurrent vitreous hemorrhage. The independent clinical and demographic variables were age, gender, DM duration, type of DM treatment, arterial hypertension, existence of UACR, eGFR, mean HbA1c and hemoglobin levels, the presence of COPD, and treatment with anticoagulant or antiplatelet drugs. The studied outpatient treatment variables were the use of ranibizumab before the first vitrectomy and the number of retinal laser photocoagulation sessions applied in each eye before the first vitrectomy. The independent surgical variables were the application of segmentation and delamination techniques, the use of diathermy, the use of silicone oil as a tamponade, and the occurrence of iatrogenic retinal tears.

Descriptive statistical analysis was performed for the quantitative data. For the qualitative data, we analyzed the frequency and percentage in each category. The normal data curve was evaluated using the Kolmogorov–Smirnov test. Differences between normally distributed quantitative variables were examined using Student’s *t*-tests; in other cases, we used Mann–Whitney U tests. Inferential analyses for qualitative data were carried out using chi-squared tests and to determine the significance of the Phi test. To correlate two continuous normally distributed quantitative variables, we used Pearson’s parametric coefficient. For categorical variables, we used Spearman’s coefficient. We used logistic regression analysis to study which independent variables were significantly related to the presence of RVH. We used multiple regression analysis to study which independent variables were significantly related to the number of vitreous hemorrhage episodes. A value of *p* < 0.05 was considered statistically significant.

## 3. Results

### 3.1. Baseline Data of the Studied Groups

Diabetes patients with PDR were recruited between 1 June 2010 and 30 June 2020. We evaluated 183 eyes from 121 T2DM patients (*n* = 126 eyes (65%) from 79 men; *n* = 57 eyes (35%) from 42 women). A total of 68 eyes (37%) had more than 1 vitreous hemorrhage episode (RVH group), while 115 eyes had only a single vitreous hemorrhage event (SVH group). The mean age of the sample was 64.12 ± 12.54 years. The mean follow-up period was 8.47 ± 1.17 years (7–21). Table 1 shows the baseline differences between the SVH and RVH groups with respect to clinical data and outpatient treatments received. In the present study, 183 eyes with PDR had vitreous hemorrhages, of which 68 eyes (37%) had RVH. The majority of eyes with RVH rebled once (61%) or twice (33%). A total of 57 eyes (84%) had late RVH, and 11 eyes (16%) had early RVH.

### 3.2. Study of Clinical Risk Factors

Table 1 shows a statistical study conducted for SVH and RVH. Women had a greater proportion of eyes with SVH while men had a greater proportion of eyes with RVH; the differences were significant with *p* = 0.002. Other attending risk factors, such as older age and longer DM duration, were substantial in the SVH group and exhibited significant differences. Patients who suffered RVH showed lower levels of hemoglobin. Regarding systemic diabetic complications, patients who had experienced more vitreous hemorrhage episodes were more likely to have suffered ischemia in the lower limbs, ischemic heart disease, and diabetic neuropathy. Among attending local eye risk factors, the presence of vitreous attached to the retina was a risk factor for RVH, and previous retina laser photocoagulation was more frequent in the SVH group than in the RVH group.

According to the bilaterality of hemorrhages, we counted fifty patients who had recurrent hemorrhage (RVH) with one or both eyes as follows: eighteen patients had bilateral involvement; in nineteen patients, only one eye RVH succeeded; in thirteen patients, they had RVH in one eye and single bleed (SVH) in the contralateral eye. Regarding the statistical differences between both eyes, they were not significant. The statistical analysis was performed with the chi-square statistic, and the results were as follows: the study of bilateral RVH according to eye significance was *p* = 0.181, the study of RVH in one eye and not with respect to contralateral significance was *p* = 0.197, and the study of RVH in one eye and SVH with respect to contralateral eye significance was *p* = 0.204.

Table 1 shows that patients in the recurrent vitreous hemorrhage group were predominantly male, younger, with longer duration of diabetes, lower hemoglobin levels, less retinal laser photocoagulation, and a higher proportion had the posterior vitreous attached to the retina in comparison with patients in the single vitreous hemorrhage group.

**Figure 1:**
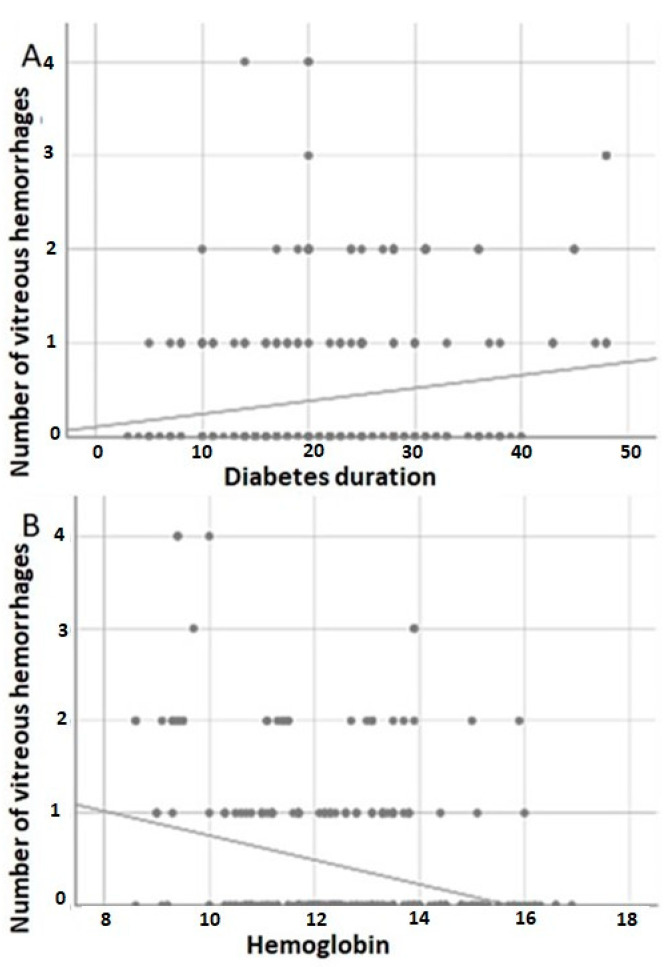
Number of vitreous hemorrhage events as a function of the duration of diabetes and the level of hemoglobin. (**A**) Scatter plot showing a positive correlation between the duration of diabetes and the number of vitreous hemorrhage events. (**B**) Scatter plot showing a negative correlation between the hemoglobin level and the number of vitreous hemorrhage episodes.

The mean levels of hemoglobin were different between the groups: SVH = 12.60 ± 1.55 and RVH = 11.71 ± 1.89 (*p* = 0.001). The lower the hemoglobin level, the more episodes of vitreous hemorrhage (r = −0.306; *p* < 0.001) (Table 2) (Figure 1).

Regarding the status of the vitreous, significant differences were observed between the groups of patients at the beginning of the study (*p* = 0.001). In 107 eyes (59%), the vitreous was initially attached to the retina, whereas 76 eyes (41%) exhibited PVD. Patients in the former group were younger (59.41 ± 12.78 years) than those with PVD (72.34 ± 10.19 years). In most eyes in the RVH group, the vitreous was initially attached to the retina, whereas in the SVH group, it was only observed in half of the eyes. The vitreous being attached to the posterior pole of the eye was found to be a risk factor for RVH (OR = 2.26, 95% CI, 1.198–4.265, *p* = 0.012) (Table 2).

Patients diagnosed with COPD were distributed homogeneously across both groups; thus, it was not linked to vitreous rebleeding. Patients on antiplatelet or anticoagulant treatment were distributed homogeneously across both groups and did not show an increased risk of presenting with eye rebleeding.

In the present study, the mean levels of HbA1c were similar in the two groups of patients; we did not observe a correlation between the level of HbA1c and the number of vitreous hemorrhage events.

Regarding the type of treatment, patients treated with oral antidiabetic drugs (17%) or insulin (83%) were similar; this did not suggest any tendency to present rebleeding. The BMI values were similar at the start of the study and did not correlate with any tendency to present vitreous hemorrhage. Regarding both the UACR and the eGFR, there were no differences between the groups at the start of the study; these factors did not suggest any significant tendency to suffer RVH.

### 3.3. Relationship with Other Diabetes Complications

The existence of systemic complications was recorded in all patients. Approximately one-third of patients (38%) with diabetic polyneuropathy developed RVH, which was a higher percentage than the other patients (*p* < 0.001). Similarly, patients diagnosed with some type of ischemia in the lower limbs (35%) were more prone to RVH when compared to the other patients (*p* < 0.001). In the same way, patients who had ischemic cardiopathy (25%) were more prone to RVH (*p* < 0.001). The rate of occurrence of stroke was similar among the groups and was not associated with eye rebleeding.

#### Study of Outpatient Retinal Treatment

We evaluated the effect of retinal laser photocoagulation and intravitreal ranibizumab, both applied before the first vitrectomy, in the prevention of recurrent vitreous hemorrhage.

### 3.4. Study of the Effect of Retinal Laser Photocoagulation

In total, 68 eyes included in the study did not receive retinal laser treatment prior to vitrectomy. On the other hand, 24, 42, 31, 14, and 4 eyes underwent 1, 2, 3, 4, and 5 laser retinal sessions, respectively. In the SVH group, 84 eyes (46%) received at least one laser session; only 31 eyes (17%) in the RVH group had undergone at least one laser session. Therefore, eyes that had previously been treated with lasers before the first surgery were less likely to bleed again (*p* < 0.0005) (Table 2).

### 3.5. Study of the Effect of Ranibizumab

A total of 43 eyes (60%) in the SVH group and 28 eyes (40%) in the RVH group previously received ranibizumab for DME. Most eyes received three injections (37.5%). A total of 71 eyes (65%) in the SVH group and 39 eyes in the RVH group had not previously been treated with ranibizumab. Prior treatment with ranibizumab therapy, independent of the number of injections, did not influence the tendency to rebleed (*p* = 0.80).

#### Study of Surgical Procedures

We recorded the differences observed with respect to the presence of tractional retinal detachment, the application of segmentation and delamination maneuvers, the use of endodiathermy and silicone oil, and the occurrence of iatrogenic retinal tears in the two groups of patients studied (Table 3).

### 3.6. Presence of Tractional Retinal Detachment

In the RVH group, 43 eyes (63%) initially showed tractional retinal detachment, of which 32 involved the macula and 11 involved the peripheral retina. On the other hand, only 5 eyes (5%) in the SVH group presented tractional retinal detachment (*p* = 0.005). Therefore, the presence of tractional retinal detachment was an independent risk factor for RVH.

### 3.7. Study of the Effect of Segmentation and Delamination

A total of 43 eyes (63%) in the RVH group and only 5 eyes (4%) in the SVH group underwent segmentation and delamination surgical maneuvers (*p* < 0.0005). In addition, the performance of these maneuvers was correlated with the occurrence of retinal tears (r = 0.84; *p* < 0.005).

### 3.8. Study of the Effect of Diathermy

Diathermy was applied to 25 eyes (22%) in the SVH group and 58 eyes (85%) in the RVH group (*p* = 0.005). This maneuver helped to avoid both intraoperative bleeding and recurrent vitreous hemorrhage events (*p* < 0.005).

### 3.9. Study of the Effect of Silicone Oil

In 42 eyes (23%), silicone oil had been used as a tamponade at the end of surgery: 29 eyes (69%) rebled, whereas 13 eyes did not rebleed. There were 141 eyes that had not received silicone oil, and only 44 eyes (31%) rebled. Significantly more eyes rebled that had received silicone oil (*p* = 0.001). The mean time at which silicone oil remained in the vitreous cavity was 3.58 ± 10.78 and 6.31 ± 9.55 months in the SVH and RVH groups, respectively (*p* = 0.078).

The eyes that presented recurrent vitreous hemorrhage rebled at 6.22 ± 2.11 [2,3,4,5,6,7,8,9,10,11,12,13] months after silicone oil was removed. The time of rebleeding did not correlate with the following variables: diabetes duration, hemoglobin level, the number of previous laser sessions, and the time silicone oil remained in the vitreous cavity.

#### Study of the Occurrence of Retinal Tears during Vitrectomy

In the RVH group, 36 eyes (53%) had at least one retinal tear during vitrectomy; this only occurred in 3 eyes (2.6%) in the SVH group (*p* < 0.0005). However, the occurrence of retinal tears was not considered a surgical risk factor for the presence of recurrent vitreous hemorrhage events.

### 3.10. Causes of Eye Rebleeding

A total of 11 eyes exhibited early RVH. In 10 eyes, rebleeding was identified within the first 24 h, of which the intraocular pressure was inferior to 10 mmHg in 7 eyes (64%). In all cases, an active residual fibrovascular membrane was observed. In total, 57 eyes presented with late RVH. In 35 eyes (61%), we observed recurrent neovascular membrane formation on the posterior pole; in 13 eyes (23%), fibrovascular ingrowth was observed at the sclerotomy sites via Doppler ultrasonography; and in 9 eyes (16%), we could not establish the cause of ocular rebleeding.

## 4. Discussion

This study included a significantly greater number of men who were more related to RVH than women. Moreover, in population-based studies of prevalence, other authors found a greater proportion of men with severe PDR. Klein et al. [16] found that men were twice as likely to suffer PDR than women in southern Wisconsin, whereas Nittala et al. [17] reported that being male and the duration of diabetes were the strongest risk factors for the development of PDR in a Latin American population. Furthermore, Hammes et al. reported that being male was a risk factor for developing T2DM in a large prospective study in Central Europe [18].

In the present study, we observed that patients with RVH had a significantly lower blood hemoglobin level. Qiao et al. [19] and Bahar et al. [20] found that DM patients with hemoglobin levels lower than 12 mg/dL were twice as likely to develop DR. In addition, Yafeng et al. found that anemia, independently of diabetic nephropathy, seems to play a significant role in the progression of DR to more severe forms [21]. It seems logical, therefore, to suppose that patients with PDR with lower hemoglobin levels aggravate their retinal hypoxia, which could then lead to the greater formation of new vessels. The etiology and pathogenesis of anemia in DM patients are multifactorial. Chronic hyperglycemia causes oxidative stress, autonomic neuropathy, and sympathetic denervation, leading to renal hypoxia and, finally, a reduction in erythropoietin production [22].

Patients with a longer duration of DM were more prone to suffering RVH. It is well known that the duration of diabetes is one of the strongest predictors of the development and progression of DR [23]. The longer the duration of diabetes, the more likely inflammatory factors are to develop, such as platelet-derived growth factor and VEGF, which have an active role in the evolution of DR and the development of new vessels [24].

The number of patients treated with anticoagulants and antiplatelet agents was similar in the two groups studied. The most used drugs were warfarin and acetylsalicylic acid. However, the role of these drugs in vitreous hemorrhage in patients with PDR is still inconclusive. Brown et al. found no differences in the incidence of vitreous hemorrhage in a group of diabetic patients who underwent vitrectomy while taking warfarin compared to the others [25]. Similarly, Frank et al. found that neither aspirin nor clopidogrel increased the risk of vitreous hemorrhage in patients with PDR. In addition, the ETDRS study demonstrated that the use of aspirin did not increase the occurrence of vitreous/preretinal hemorrhages in patients with diabetes who require it for the treatment of cardiovascular disease or other medical indications [26]. In contrast, Talany et al. carried out a retrospective study based on a review of all ocular events registered in the Vigibase database of the World Health Organization that were related to the use of warfarin and new oral anticoagulants, and they concluded that the risk of intraocular hemorrhage is higher in the latter group [27].

In the present study, for most eyes in the RVH group, the vitreous was initially attached to the retina as opposed to the SVH group in which only half of the eyes had the vitreous attached. Although the posterior hyaloid was removed in the first vitreoretinal surgery in eyes that bled and retinal laser photocoagulation was completed, the likelihood of peripheral vitreous remnants being attached to the retina in addition to retinal ischemia might facilitate the growth of new vessels and the occurrence of subsequent episodes of vitreous hemorrhage in some eyes.

Patients in our study with RVH had more myocardial and lower limb ischemia compared to the SVH group. However, the incidence of stroke was similar among them. Zhu et al. [28] found a graded relationship between the severity of DR and the risk of all-cause mortality in patients with T2DM mainly due to heart failure and stroke, which is indicative that PDR individuals exhibit more risk factors than those with non-proliferative DR. Similarly, in a prospective cohort study, Cheung et al. [29] found that DR was an independent risk factor for ischemic stroke. Other studies were inconclusive, however, regarding the association between DR and stroke [30]. The UKPDS [5] reported that DR was not a significant risk factor for stroke, and the Wisconsin Epidemiological Study of Diabetic Retinopathy concluded that only PDR and not NPDR was associated with stroke in T2DM patients [31]. Unlike non-diabetic patients, the type of stroke in diabetic patients mainly affects microvascular circulation instead of large vessels [32,33].

In our study, eyes that had previously undergone retinal laser photocoagulation had fewer vitreous hemorrhage events. The greater number of retinal sessions applied to the eyes, the fewer episodes of vitreous hemorrhage they presented. Patients with PDR who underwent PRP exhibited reductions in their vitreous levels of VEGF, thus reducing future risk of vitreous hemorrhage [34]. On the other hand, we observed that the tendency to rebleed was similar regardless of whether or not the eyes had been treated with ranibizumab. Treated eyes received only a mean of three ranibizumab injections, which could explain the lack of anti-VEGF effects observed.

A quarter of the eyes initially showed a tractional retinal detachment, most of which involved the macula. The macula represented a large proportion of the total number of eyes with PDR. One possible explanation is that our reference diabetic population was mostly of rural origin, and a significant proportion of patients usually attended the ophthalmology service once DR became noticeable. For this reason, it is very common to visit patients with multiple fibrovascular proliferations in both eyes who have not previously received retinal laser photocoagulation. All eyes with tractional retinal detachment underwent segmentation and delamination by bimanual surgery, of which half of them experienced RVH. Eyes with tractional retinal detachment show higher levels of VEGF, which facilitate the growth of new vessels [35]. Intra-surgical diathermy was used in most cases that required segmentation and delamination, while it was only performed in 17% of those that did not. This procedure significantly reduced the tendency to re-bleed in the long term.

Silicone oil was used as a tamponade in only 23% of eyes to minimize the negative retinal effects associated with its long-term use, such as thinning of the nerve fiber layer, decreased choroidal thickness, and alterations in retinal microcirculation [36,37]. The time during which silicone oil remained in the vitreous cavity in the SVH and RVH groups was 3.5 and 6.3 months, respectively. However, two-thirds of the eyes in which silicone oil was used experienced late RVH. We hypothesize that the reason might be because silicone oil is reserved for the most complicated eyes, and although no eyes rebled when treated with silicone oil, some rebled after it was removed.

In our study, only 8% of eyes in which no silicone oil was used (141 eyes) showed early RVH. This was achieved by avoiding the postoperative low intraocular pressure that only six eyes suffered. The vitreoretinal surgeons of our service sutured the sclerotomies in patients with PDR to avoid postoperative ocular hypotony. On the other hand, 57 eyes presented with late RVH. The most frequently observed causes of late rebleeding were persistent or recurrent neovascular membrane formation (35 eyes, 61%) at the posterior pole and fibrovascular growth at sclerotomy sites (13 eyes, 18%) observed by Doppler ultrasonography. West et al. found that fibrovascular ingrowth was the main cause of rebleeding after diabetic vitrectomy [38], whereas for Shi et al., it was the presence of residual fibrovascular membranes and new vessel growth [39].

To the best of our knowledge, this is the first study aimed at evaluating clinical, epidemiological, and surgical differences between patients with PDR whose eyes did not bleed once posterior vitrectomy was carried out and in whom PRP was usually completed compared with patients who tended to rebleed despite undergoing vitrectomy and retinal laser photocoagulation. The limitations of our study were its retrospective design, the small sample size, and the fact that the surgeries were performed by different vitreoretinal surgeons.

In the present study, we observed that both clinical and surgical variables were related to the presence of multiple vitreous hemorrhage events.

## 5. Conclusions

Our study showed that the patients with PDR who are the most prone to eye rebleeding are men who have a longer duration of diabetes, anemia, the vitreous is attached to the retina, they have not undergone retinal laser photocoagulation, and they have previously suffered a cardiovascular event. Regarding surgical variables, while the presence of tractional retinal detachment increases the risk of RVH, the use of intraoperative diathermy reduces its future occurrence. Therefore, the treatment of anemia and early application of retinal laser photocoagulation are recommended to reduce the likelihood of rebleeding. In the same way, intraoperative diathermy is recommended to avoid RVH. However, due to the limitations of this study, more research is needed to confirm these findings.

## Figures and Tables

**Table 1:** Statistical study of differences between SVH and RVH groups of patients. UACR, urine albumin to creatinine ratio; eGFR, estimated glomerular filtration rate; COPD, chronic obstructive pulmonary disease; PRP, panretinal photocoagulation; Men ^†^ percentage with respect to all men of the sample, Women ^‡^ percentage with respect to all women of the sample; PVD patients with posterior vitreous detachment. *, chi-squared test; **, Mann–Whitney U test; ***, Student’s *t*-test. Phi^+^, Mathew’s correlation coefficient. F^++^, Fisher–Snedecor.

Variable	Single Vitreous Hemorrhage (SVH)	Recurrent Vitreous Hemorrhage (RVH)	Significance
Gender:			Phi^+^ = 0.180, *p* = 0.002 *
Men ^†^	38 (48%)	41 (51%)	
Women ^‡^	33 (78%)	9 (22%)	
Age (years)	66.08 ± 11.81	62.59 ± 15.51	F^++^ = 4.131, *p* = 0.017 **
	(28–88)	(34–91)	
DM duration (years)	22.02 ± 9.30	24.35 ± 10.65	F = 2.721, *p* = 0.043 **
	(20.3–23.7)	(21.7–26.9)	
Arterial hypertension	21 (29.5%)	8 (15.1%)	Phi = 0.083, *p* = 0.37 *
DM treatment:			
Insulin	57 (81%)	43 (87%)	Phi = 0.082, *p* = 0.38 *
Oral drugs	14 (19%)	7 (13%)	Phi = 0.079, *p* = 0.41 *
Anticoagulant users	18 (18.5%)	18 (36%)	Phi = 0.08, *p* = 0.058 *
Antiplatelet users	59 (20.7%)	37 (13%)	Phi = 0.093, *p* = 0.295 *
No Previous PRP	31 (27%)	37 (54%)	Phi = −0.275, *p* = 0.005 *
Previous PRP	84 (73%)	31 (46%)	
No COPD	84 (80%)	44 (44.7%)	Phi = 0.088, *p* = 0.23 *
COPD	31 (20%)	24 (20.3%)	
Hemoglobin (g/dL)	12.60 ± 1.55	11.71 ± 1.89	F = 5.69, *p* = 0.001 **
	(12.3–12.8)	(11.2–12.1)	
Mean HbA1c	8.81 ± 1.61%	8.88 ± 1.77%	F = 0.004, *p* = 0.773 ***
	(8.5–9.1)	(8.4–9.3)	
No UACR	39 (55%)	22 (45%)	Phi = 0.120, *p* = 0.165 *
UACR	32 (44%)	28 (56%)	
eGFR (mL/min/1.73^2^)	66.25 ± 25.66	60.63 ± 25.55	F = 0.361, *p* = 0.147 **
	(61.51–70.99)	(54.71–66.55)	
Phakic	70 (61%)	40 (59%)	Phi = 0.020, *p* = 0.785 *
Pseudophakic	45 (39%)	28 (41%)	
No PVD	57 (49%)	50 (73%)	Phi = −0.235, *p* = 0.001 *
PVD	58 (51%)	18 (27%)	
Previous therapy Ranibizumab	71 (62%)	39 (57%)	Phi = 0.049, *p* = 0.804 *
	43 (38%)	28 (43%)	

**Table 2:** Logistic regression study of each independent variable in relation to the recurrence of vitreous hemorrhage (RVH), assessed by means of logistic regression analysis. The gender, duration of diabetes, level of hemoglobin, presence of the posterior vitreous attached to the retina, number of retinal laser sessions before the first episode of vitreous hemorrhage, and presence of tractional retinal detachment were risk factors in our population of diabetic patients with proliferative diabetic retinopathy who showed recurrent vitreous hemorrhage. On the other hand, intraoperative diathermy significantly reduced the occurrence of RVH and was also significant.

Independent Variables	B	CI 95%	Significance
Gender	0.082	0.016–0.423	*p* = 0.003
Age	0.950	0.893–1.010	*p* = 0.101
DM duration	1.915	1.295–3.219	*p* = 0.028
Body mass index	0.927	0.821–1.046	*p* = 0.216
Arterial hypertension	0.731	0.51–0.851	*p* = 0.209
DM treatment	1.094	0.411–3.674	*p* = 0.392
Anticoagulant therapy	0.769	0.137–2.308	*p* = 0.765
Antiplatelet therapy	1.180	0.567–2.380	*p* = 0.257
Number of retinal laser sessions	2.931	1.096–4.734	*p* = 0.01
COPD	0.488	0.226–1.201	*p* = 0.533
Hemoglobin	2.439	1.434–3.942	*p* = 0.014
HbA1c	1.229	0.863–1.751	*p* = 0.252
UACR	0.249	0.098–1.829	*p* = 0.249
eGFR	0.991	0.966–1.017	*p* = 0.516
Previous anti-VEGF therapy	1.065	0.814–2.457	*p* = 0.110
Posterior vitreous detachment	2.261	1.131–4.420	*p* = 0.012
Traccional retinal detachment	4.022	1.007–7.07	*p* < 0.001
Intraoperative diathermy	3.259	1.057–6.184	*p* < 0.001
Retinal tear	1.028	0.087–1.713	*p* = 0.09

**Table 3:** Differences in surgical variables between the two groups of patients studied with proliferative diabetic retinopathy. Eyes in the recurrent vitreous hemorrhage (RVH) group were more likely to have a tractional retinal detachment in which segmentation and delamination were applied, silicone oil was used more frequently, and more retinal tears were observed during the vitrectomy. * Chi-squared test.

Variable	SVH	RVH	Significance
Tractional retinal detachment			Phi = 0.648, *p* = 0.005 *
No	110 (95%)	25 (37%)	
Macular detachment	3 (3%)	32 (47%)	
Peripheral detachment	2 (2%)	11 (16%)	
Segmentation/Delamination			Phi = 0.647, *p* = 0.005 *
Yes	5 (4%)	43 (63%)	
No	110 (96%)	25 (37%)	
Endodiathermy			Phi = 0.617, *p* < 0.005 *
Yes	25 (22%)	58 (85%)	
No	90 (78%)	10 (15%)	
Silicone oil			Phi = 0.306, *p* = 0.005 *
Yes	15 (13%)	27 (40%)	
No	100 (87%)	41 (60%)	
Occurrence of retinal tears			Phi = 0.594, *p* = 0.005 *
Yes	3 (3%)	36 (57%)	
No	112 (97%)	32 (43%)	

## Data Availability

The datasets used and/or analyzed during the current study are available from the corresponding author upon reasonable request.

## Abbreviations

RVH	recurrent vitreous hemorrhage
PDR	proliferative diabetic retinopathy
BMI	body mass index
UACR	urine albumin to creatinine ratio
eGFR	estimated glomerular filtration rate
DR	diabetic retinopathy
DM	diabetes mellitus
DME	diabetic macular edema
DCCT	Diabetes Control and Complications Trial
UKPDS	United Kingdom Prospective Diabetes Study
T1DM	type 1 diabetes mellitus
T2DM	type 2 diabetes mellitus
PRP	panretinal photocoagulation
PVD	posterior vitreous detachment
SVH	single vitreous hemorrhage
PPV	pars plana vitreoretinal surgery
OCT	optical coherence tomography
FA	fluorescein angiography
COPD	chronic obstructive pulmonary disease
VEGF	vascular endothelial growth factor
